# Surgical resection of a lipoma of the right atrium: A case report

**DOI:** 10.1097/MD.0000000000041848

**Published:** 2025-03-21

**Authors:** Yifan Yan, Chen Ma, Bhushan Sandeep, Xiufang Su, Zongwei Xiao

**Affiliations:** a Department of Cardio-Thoracic Surgery, Chengdu Second People’s Hospital, Chengdu, Sichuan, China.

**Keywords:** benign tumor, lipoma, right atrium, surgical resection

## Abstract

**Rationale:**

Cardiac tumors are relatively rare, with an incidence rate of 0.17% to 0.19% according to autopsy reports. The tumors may be detected through transthoracic echocardiography, computed tomography (CT), magnetic resonance imaging (MRI), and other examinations. The treatment plan is determined based on the nature of the tumor.

**Patient concerns:**

In this article, we report a case of a 61-year-old male who was found to have an occupying lesion in the right atrium and superior vena cava during a cardiac ultrasound examination.

**Diagnoses:**

Enhanced CT and MRI suggested the possibility of a lipoma during admission.

**Interventions:**

The patient underwent video-assisted thoracoscopic cardiac tumor resection under extracorporeal circulation. The postoperative pathology was consistent with lipoma.

**Outcomes:**

The recurrence rate after cardiac lipoma excision is low, and the prognosis is generally good. However, for patients with cardiac lipomas involving ventricles and myocardial infiltration, intraoperative excision is more challenging, and the long-term outcome is poor.

**Lessons:**

Cardiac lipomas are generally asymptomatic even in large dimensions. Echocardiograms can identify tumors, but cardiac MRI or cardiac CT can differentiate cardiac lipomas from other cardiac tumors.

## 1. Introduction

Cardiac tumors are relatively rare, with an incidence rate of 0.17 to 0.19% according to autopsy reports.^[1]^ They are classified into primary tumors and metastatic tumors, with cardiac metastases being more common than primary tumors. Cardiac tumors can cause related symptoms (such as pulmonary embolism, systemic embolism, heart failure, valvular regurgitation, arrhythmias, conduction blocks, and cardiac tamponade).^[2]^ Tumors in the right atrium may present with hemodynamic changes similar to tricuspid valve disease, including symptoms related to right heart failure, such as peripheral edema, hepatosplenomegaly, ascites, jugular venous distension, and in some patients, a diastolic murmur may be auscultated. However, some patients may be asymptomatic, and the tumors may be detected through transthoracic echocardiography, computed tomography (CT), magnetic resonance imaging (MRI), and other examinations. The treatment plan is determined based on the nature of the tumor.

## 2. Case presentation

**Figure 1.**
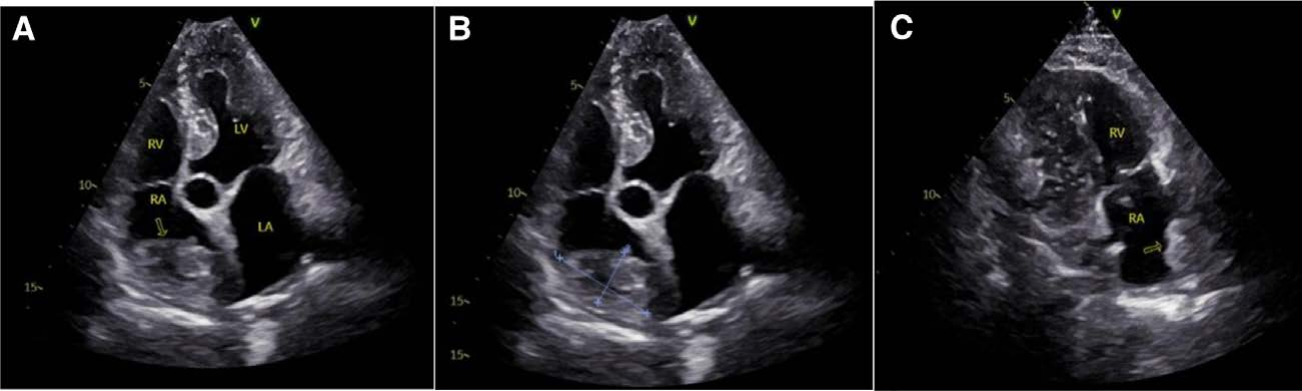
(A) A moderately echogenic mass is visible in the right atrium; (B) a medium-sized echo cluster measuring approximately 21 × 28 × 48 mm was detected near the roof in the right chamber; (C) the mass extends from the right atrium to the superior vena cava, with a wide base and an unclear border with the atrial wall. No obvious activity or color flow signal is observed.

**Figure 2.**
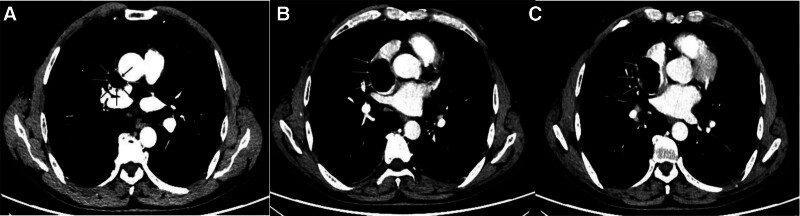
(A) A low-density mass is visible at the junction of the superior vena cava and the right atrium; (B) the mass has an intact capsule with clear boundaries, measuring approximately 4.1 × 2.8 cm, with a CT value of −91 HU; (C) There appears to be internal septation, and no enhancement is observed on enhanced scanning. CT = computed tomography.

**Figure 3.**
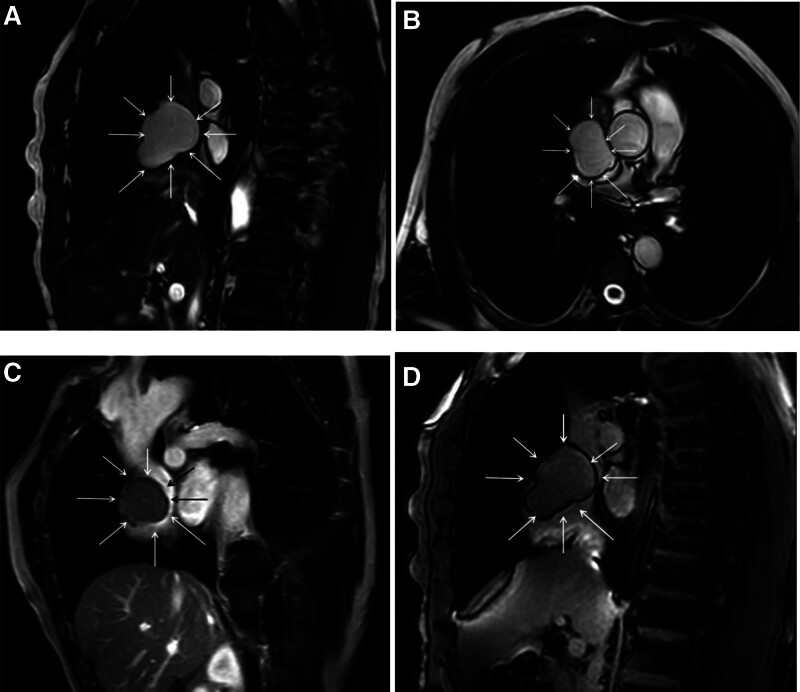
(A) Visible signal shadow in the superior vena cava and right atrium, with stenosis at the entrance of the superior vena cava; (B) It can be seen that the capsule of the mass is intact, the boundary is clear, and the size is about 4.8 × 3.8 × 3.4 cm; (C, D) No significant enhancement of the mass was observed.

The patient is a 61-year-old male who was found to have an occupying lesion in the right atrium and superior vena cava during a cardiac ultrasound examination 20 days prior to admission. He had no abnormal physical signs and a history of type 2 diabetes. The echocardiogram (Fig. 1) suggested: a medium echogenic mass in the right atrium and superior vena cava, extending into the superior vena cava, with a broad base and indistinct margins from the atrial wall, showing no significant mobility. Contrast-enhanced chest CT (Fig. 2): a mass with low density was seen in the lumen of the superior vena cava-right atrium, with clear boundaries and no enhancement on enhanced scanning. A lipoma was suspected. Cardiac MRI (Fig. 3): a lipoma in the right atrial area with narrowing of the superior vena cava inlet. Coronary angiography: no calcification or stenosis was seen in the left and right coronary arteries. Other examinations showed no abnormalities.

### 2.1. Treatment plan

**Figure 4.**
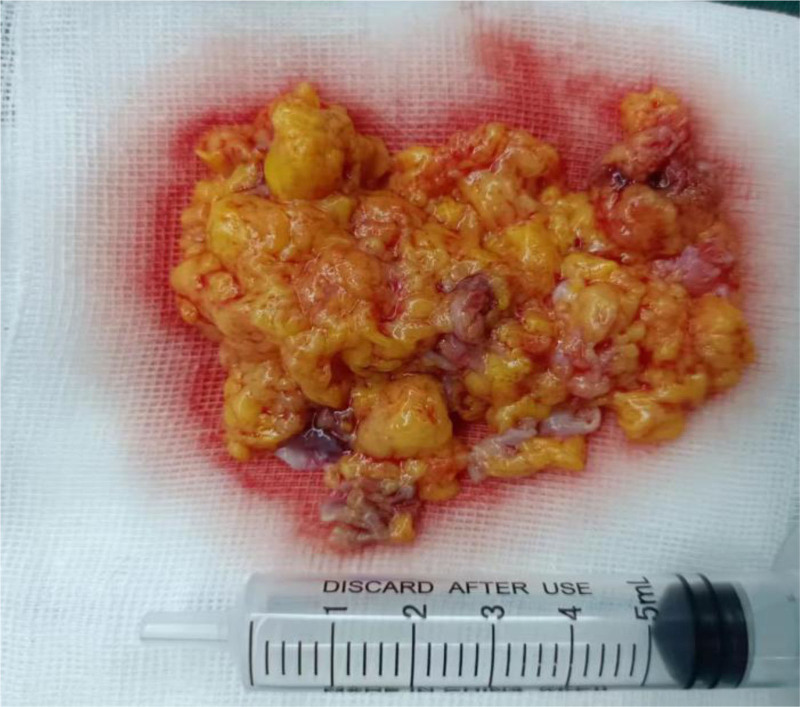
Gross surgical specimen: a large amount of fatty tissue is visible.

**Figure 5.**
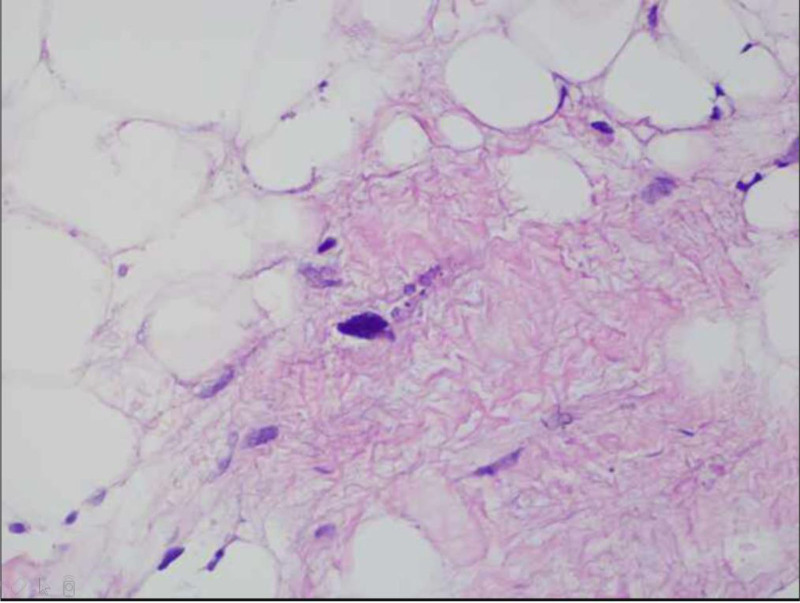
Postoperative pathological results: lipomatous and rhabdomyomatous hyperplasia-like proliferation, with some adipocytes showing atypia and widespread enlargement of rhabdomyocytes with deep staining. Lipoma is considered.

Four days after admission, the patient underwent video-assisted thoracoscopic resection of the cardiac tumor under extracorporeal circulation. The second intercostal space along the anterior axillary line and the fourth intercostal space were used as operating ports, and the fifth intercostal space along the mid-axillary line was used as the observation port. During the surgery, a tumor about 5cm by 4cm in size, pale yellow in color, soft in texture, and with an intact capsule was observed in the right atrium. Part of the tumor had extended into the opening of the superior vena cava. The tumor was gradually separated from the inner walls of the right atrium and the superior vena cava, and the atrial tumor was excised (Fig. 4). Exploration of the atrium and superior vena cava revealed no significant residual tumor, and the incision was closed. The pathology report (Fig. 5) described lipomatous and rhabdomyomatous tumor-like hyperplasia, with some lipocytes showing atypia and rhabdomyocytes extensively enlarged and deeply stained. A lipoma was considered. Echocardiograms performed on the day of surgery and 4 months postoperatively showed no significant abnormalities in cardiac structure and blood flow, with normal left ventricular systolic function measurements.

## 3. Discussion

Cardiac lipomas are a type of benign cardiac tumor, primarily composed of benign adipose cells and characterized by a complete fibrous capsule.^[3,4]^ Cardiac lipomas usually have clear boundaries, but in cases where the lipoma invades the myocardium or other tissues, the boundaries may be indistinct. Cardiac lipomas can cause related symptoms, and the tumor mass may grow progressively, thus necessitating surgical excision.^[5,6]^ Preoperative routine assessment for cardiac tumors includes echocardiography, cardiac MRI, cardiac CT, and coronary angiography, especially coronary angiography to assess the tumor’s feeding vessels and the extent of vascular invasion by the tumor. Transesophageal echocardiography (TEE) provides better imaging due to its proximity to the left atrium. Cardiac CT can differentiate tissues based on CT values, with adipose tissue having CT values ranging from −80 to −100 HU, which allows it to be distinguished from myocardium and thrombi, as well as myxomas.^[7]^ Cardiac MRI not only shows detailed structures but also T1 and T2 weighted sequences can reflect different tissue types. CT and MRI are highly specific in identifying fat.^[8–11]^ The drawbacks of cardiac MRI are its high equipment cost and examination fees, and it requires the absence of metallic foreign bodies in the body, which may not be suitable for all patients. Cardiac lipomas can be aided in diagnosis by the characteristic images displayed by echocardiography and MRI. Pathological biopsy after surgical excision can clarify the exact tissue type of the tumor. The recurrence rate after cardiac lipoma excision is low, and the prognosis is generally good.^[4,12]^ However, for patients with cardiac lipomas involving the ventricles and myocardial infiltration, intraoperative excision is more challenging, and the long-term outcome is poorer.^[11]^

## 4. Conclusion

Cardiac lipomas are a benign form of cardiac tumor, and some patients may be diagnosed due to related symptoms or during routine medical examinations. They are often detected through cardiac ultrasound, CT, and MRI. MRI, in particular, holds diagnostic value for lipomas because it can reflect different tissue types through T1 and T2 weighted sequences. For asymptomatic patients with larger lipomas, surgery should be considered.

## Author contributions

**Conceptualization:** Yifan Yan, Chen Ma, Bhushan Sandeep, Xiufang Su, Zongwei Xiao.

**Data curation:** Yifan Yan, Chen Ma, Bhushan Sandeep, Zongwei Xiao.

**Formal analysis:** Yifan Yan, Chen Ma, Bhushan Sandeep, Zongwei Xiao.

**Investigation:** Yifan Yan, Chen Ma, Bhushan Sandeep, Xiufang Su, Zongwei Xiao.

**Methodology:** Yifan Yan, Bhushan Sandeep, Zongwei Xiao.

**Project administration:** Yifan Yan, Chen Ma, Bhushan Sandeep, Xiufang Su, Zongwei Xiao.

**Resources:** Yifan Yan, Bhushan Sandeep, Xiufang Su, Zongwei Xiao.

**Software:** Yifan Yan, Bhushan Sandeep, Zongwei Xiao.

**Supervision:** Yifan Yan, Chen Ma, Bhushan Sandeep, Xiufang Su, Zongwei Xiao.

**Validation:** Yifan Yan, Chen Ma, Bhushan Sandeep, Xiufang Su, Zongwei Xiao.

**Visualization:** Yifan Yan, Chen Ma, Bhushan Sandeep, Xiufang Su, Zongwei Xiao.

**Writing – original draft:** Yifan Yan, Chen Ma, Bhushan Sandeep, Xiufang Su, Zongwei Xiao.

**Writing – review & editing:** Yifan Yan, Chen Ma, Bhushan Sandeep, Xiufang Su, Zongwei Xiao.
